# Optimizing Treatment Strategy for Oligometastases/Oligo-Recurrence of Colorectal Cancer

**DOI:** 10.3390/cancers16010142

**Published:** 2023-12-27

**Authors:** Ryoma Yokoi, Jesse Yu Tajima, Masahiro Fukada, Hirokatsu Hayashi, Masashi Kuno, Ryuichi Asai, Yuta Sato, Itaru Yasufuku, Shigeru Kiyama, Yoshihiro Tanaka, Katsutoshi Murase, Nobuhisa Matsuhashi

**Affiliations:** Department of Gastroenterological Surgery and Pediatric Surgery, Gifu University Graduate School of Medicine, 1-1 Yanagido, Gifu City 501-1194, Gifu, Japan; yokoi.ryoma.h1@f.gifu-u.ac.jp (R.Y.); murase.katsutoshi.f1@f.gifu-u.ac.jp (K.M.)

**Keywords:** colorectal cancer, oligometastases, oligo-recurrence, surgery, percutaneous ablation, stereotactic body radiotherapy, systemic therapy

## Abstract

**Simple Summary:**

Colorectal cancer is a prevalent disease, often progressing to metastatic stages. A subgroup of patients displaying oligometastases presents a unique opportunity for extended survival due to the advances in multidisciplinary treatment. The imperative goal is complete metastatic eradication, with surgical resection serving as the standard of care. For patients ineligible for surgery, percutaneous ablation or stereotactic body radiotherapy are viable options. Although clinical trial evidence supports perioperative systemic therapy in improving progression-free survival, consistent enhancement in overall survival is not observed. Consequently, tailored treatment strategies, considering various oncological factors, are essential for patients with oligometastatic colorectal cancer. Further prospective trials are crucial to establish a comprehensive framework for defining and optimizing the treatment strategy for oligometastatic colorectal cancer.

**Abstract:**

Colorectal cancer (CRC) is the third most common cancer, and nearly half of CRC patients experience metastases. Oligometastatic CRC represents a distinct clinical state characterized by limited metastatic involvement, demonstrating a less aggressive nature and potentially improved survival with multidisciplinary treatment. However, the varied clinical scenarios giving rise to oligometastases necessitate a precise definition, considering primary tumor status and oncological factors, to optimize treatment strategies. This review delineates the concepts of oligometastatic CRC, encompassing oligo-recurrence, where the primary tumor is under control, resulting in a more favorable prognosis. A comprehensive examination of multidisciplinary treatment with local treatments and systemic therapy is provided. The overarching objective in managing oligometastatic CRC is the complete eradication of metastases, offering prospects of a cure. Essential to this management approach are local treatments, with surgical resection serving as the standard of care. Percutaneous ablation and stereotactic body radiotherapy present less invasive alternatives for lesions unsuitable for surgery, demonstrating efficacy in select cases. Perioperative systemic therapy, aiming to control micrometastatic disease and enhance local treatment effectiveness, has shown improvements in progression-free survival through clinical trials. However, the extension of overall survival remains variable. The review emphasizes the need for further prospective trials to establish a cohesive definition and an optimized treatment strategy for oligometastatic CRC.

## 1. Introduction

Colorectal cancer (CRC) is the third most common cancer and the second leading cause of cancer-related death worldwide [1]. Approximately 50% of CRC patients develop metastatic disease, and about a half of them develop metachronously after treatment for locoregional CRC [2,3]. The most common site of metastasis is the liver, and then lung, peritoneum, and distant lymph nodes. Metastasis to the bone, brain, and other sites is infrequent [4,5]. The 5-year relative survival for all stages is 65.0%. Meanwhile, for stage IV (metastatic CRC: mCRC), that rate drops to 15.6%, although the survival for mCRC patients has been extended because of the advances in multidisciplinary treatment strategies including systemic therapy and local treatment for primary and metastatic disease [6]. Various oncological factors can impact survival for patients with mCRC. Synchronous colorectal liver metastasis (CRLM) is associated with a more disseminated disease status (more metastatic lesions and bilobarly distributed metastases) and a worse prognosis than metachronous CRLM [7]. Extrahepatic metastases, more than three tumors, and a disease-free interval of less than 12 months have been also reported as poor prognostic factors for patients with mCRC [8,9,10,11,12,13]. Furthermore, 5-year survival rates are low in patients with CRLM not undergoing surgery, and the majority of the mCRC patients are unsuitable candidates for resection [14,15].

On the other hand, selected patients with limited metastases can expect prolonged survival. As the contrast to widespread metastases involving multiple sites (polymetastatic disease), the concept of oligometastases, which exhibit a less aggressive form with involvement of fewer sites, was introduced by Hellman and Weichselbaum [16]. The patients with oligometastatic CRC have a good prognosis, even cure, since the disease is both locally and systemically well controlled with local treatments and systemic therapies, especially when treatment approaches for individual mCRC patients are discussed within a multidisciplinary team of experts in the field [17,18]. In fact, the median survival of patients with oligometastatic CRC is generally better (about 40 months) compared to that of patients with poly-metastatic CRC (about 20 months) [19,20]. It is important for the multidisciplinary team to play an ongoing role throughout the clinical course of the mCRC patient, initially to review the diagnostic work-up to define curability by local treatments and to consider the multidisciplinary management [18]. Therefore, the reasonable definition and classification of oligometastatic CRC are highly important to determine the optimal treatment strategy, but the diagnosis is based solely on imaging findings and no biomarker for the identification is clinically available. Although there is consensus on many aspects of oligometastatic CRC, the detailed criteria and treatment strategies have not yet been completely established and are different among studies, specialties, and clinicians [21]. Furthermore, only a few comprehensive reviews on the definition and treatment of oligometastatic CRC have been reported [22].

In this review, we summarize the concepts and diagnostic criteria for oligometastatic CRC, and then describe the key points of multidisciplinary treatment strategies with local treatments and systemic therapy for patients with oligometastatic CRC with heterogenous spectrum. For a comprehensive review, a literature search of the PubMed was performed using different combinations of terms related to ‘oligometastases’, ‘oligo-recurrence’, ‘stage IV CRC’, ‘oligometastatic CRC’, ‘local treatment for mCRC’, and ‘systemic therapy for mCRC’ in “All fields”. Current guidelines written by major cancer societies, including the National Comprehensive Cancer Network (NCCN) [23], the European Society of Medical Oncology (ESMO) [18,24,25], and the Japanese Society for Cancer of the Colon and Rectum [26], were also reviewed. The references of the included studies were screened to identify additional studies that were incorporated as appropriate.

## 2. Concept of Oligometastases/Oligo-Recurrence of CRC

In 1995, Hellman and Weichselbaum proposed the concept of “oligometastases” [16]. This was the first description of oligometastases and related concepts. Oligometastases is the state in which the patient shows distant relapse in only a limited number of regions, which can be treated by local treatments. However, their concept did not eliminate the uncontrolled primary site. Niibe et al. proposed the concept of “oligo-recurrence” as the best prognostic state in which patients had a controlled primary tumor and 1–5 metachronous metastases that could be treated with local treatment with or without systemic therapy. Then, they also classified sync-oligometastases, which had an active but controllable primary lesion, and showed that the most important prognostic factor of oligometastases was the status of the primary lesion [27,28,29].

**Figure 1:**
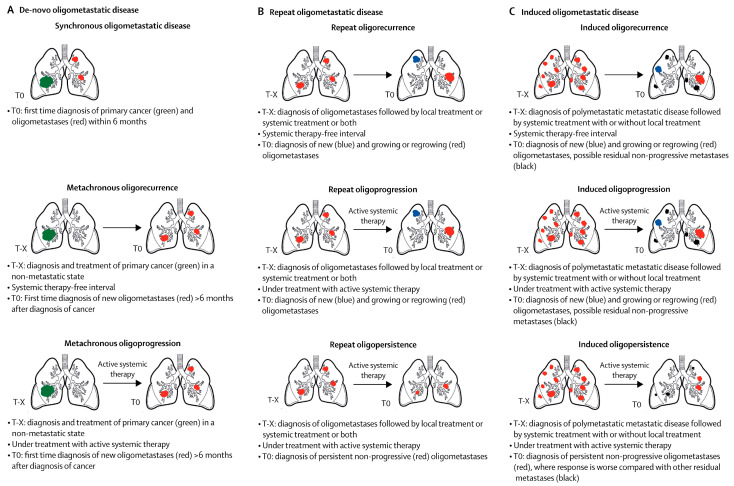
Oligometastatic disease classification system developed by European Society for Radiotherapy and Oncology and European Organisation for Research and Treatment of Cancer. T0 = at this current point of time. T-x = any previous point in time. Reprinted with permission from ref. [30]. Copyright Elsevier (2023).

Recently, European Society for Radiotherapy and Oncology (ESTRO) and European Organisation for Research and Treatment of Cancer (EORTC) have developed a classification system that differentiated nine distinct states of oligometastatic disease (Figure 1) [30]. First, the authors distinguished between “genuine” oligometastatic disease (no history of polymetastatic disease) and “induced” oligometastatic disease (previous history of polymetastatic disease) that did not indicate a possible low metastatic capacity. Next, genuine oligometastatic disease was subclassified into “repeat” oligometastatic disease (previous history of oligometastatic disease) and “de-novo” oligometastatic disease (first-time diagnosis of oligometastatic disease). Repeat and induced oligometastatic disease is assumed to have a controlled primary tumor, while de-novo oligometastatic disease is independent of whether the primary tumor is active or controlled. In de-novo oligometastatic disease, synchronous and metachronous oligometastatic disease were differentiated (maximum 6 months interval or more than 6 months interval between diagnosis of oligometastatic disease and primary cancer diagnosis). Finally, oligorecurrence, oligoprogression, and oligopersistence were subclassified, considering diagnosed timing (during a treatment-free interval or during active systemic therapy) and disease progression. This classification system will be useful for assessing the prognosis of oligometastatic disease and contribute to clinical trials and their outcome analyses. Although several oncological situations may be overlapped, classification of oligometastases are summarized in Table 1. For example, oligometastases first-time diagnosed during adjuvant chemotherapy after primary resection and during preoperative treatment 6 months after diagnosis of primary tumor are classified to oligo-recurrence and sync-oligometastases, respectively, according to Niibe et al., but both metastases can be classified to metachronous oligoprogression according to ESTRO and EORTC classification. Repeat oligoprogression/oligopersistence and induced oligometastatic disease may also be a little difficult to accommodate in classification other than that of ESTRO and EORTC.

According to current ESMO guideline, a clinical definition of oligometastatic CRC is:

- One to five metastatic lesions, occasionally more if complete eradication is possible.
- Up to two metastatic sites.
- Controlled primary tumor (optionally resected).
- All metastatic sites must be safely treatable by local treatments.

Furthermore, it is necessary to comprehensively consider including relevant factors: disease-related factors such as tumor size, burden, location, and previous oncological history, surgery and other local treatment-related factors, and patient-related factors. However, the definition does not consider biological factors and the maximum size or volume of individual metastases [18]. ESTRO and American Society for Radiation Oncology (ASTRO) consensus regarding definition of oligometastatic disease is similar to that of ESMO guideline because of the lack of clinical evidence that supports biomarkers and fixing maximum number or size of metastases to define oligometastatic disease. From a pragmatic point of view, up to 3–5 lesions and a maximum cut off size of 5–7 cm are the most commonly-used quantitative definitions, but more and larger lesions may be treatable depending on location and with careful attention [31].

On the other hand, the NCCN guidelines barely mention the concept of oligometastases, and instead describe the treatment strategy in terms of resectable or unresectable mCRC, as well as synchronous or metachronous mCRC [23]. In fact, many clinical trials have also used the main criteria of resectable mCRC. Resectable mCRC would have been used from a surgical aspect, while oligometastatic CRC in terms of more local treatments other than surgery for less aggressive and smaller metastases, such as percutaneous ablation and radiotherapy.

## 3. Local Treatments

The goal of local treatments for oligometastatic CRC is to achieve complete eradication of all metastases. The reported 5-year overall survival (OS) after CRLM resection is up to 40–50%, and even 71% if limited to solitary cases [32,33,34,35]. To prolong the time until systemic therapy is needed for polymetastatic disease and thereby maintain the patient’s quality of life could be another goal [30]. Although the optimal local treatment is controversial due to no randomized trials comparing surgical with non-surgical management, the standard of care for patients with oligometastatic CRC has remained surgical resection. If patients are unsuitable candidates for resection because of patient factors or tumor characteristics such as tumor location or insufficient organ reserve, percutaneous ablation or stereotactic body radiotherapy (SBRT) can be a reasonable option for small metastases in combination with surgery or alone [36,37,38,39,40]. In particular, emerging data suggest that SBRT is effective for local control and survival. For selected patients with more extensive liver disease that cannot be addressed with even ablation or SBRT, intra-arterial therapies, such as hepatic arterial infusion chemotherapy, transarterial chemoembolization, or transarterial radioembolization may contribute to a long-term survival or prolonged time to tumor progression, but are rarely curative [18,22,23,41,42,43,44]. These intra-arterial therapies should be considered as treatment options with non-curative intent, or in combination with other local treatments. Ultimately, clinical decision-making requires a multidisciplinary discussion that should take into account individual patient characteristics and clinical expertise available at the treatment facility.

In this section, we describe surgery, percutaneous ablation, and SBRT as main local treatments for liver and lung oligometastases of CRC, and then local treatments for peritoneal metastases.

### 3.1. Liver Metastases

#### 3.1.1. Surgery

R0 complete resection is necessary for CRLMs while maintaining sufficient liver volume to prevent post-hepatectomy liver failure. Future liver remnant should be more than 20% of the estimated liver volume in patients with a normal liver, 30% in patients with chemotherapy-induced liver injury, and at least 40% in patients with cirrhosis [45]. Furthermore, it is important to consider repeat local treatment for recurrent CRLMs since recurrences after resection for initial CRLMs occur in up to 70% of patients [46]. 5-year OS and 5-year progression-free survival (PFS) for patients undergoing a second hepatectomy for recurrent CRLMs were reported to be comparative with those for patients after an initial hepatectomy, or even better survival in selected patients with feasibility and low morbidity, although survival decreased with each subsequent surgery [47,48,49,50,51,52]. A meta-analysis found that patients meeting the following six predictors derived significant survival benefit from a repeat hepatectomy: disease-free interval after initial hepatectomy > 1 year; solitary, unilobar, or smaller than 5 cm CRLM at second hepatectomy; lack of extrahepatic metastases at second hepatectomy; and R0 resection at second hepatectomy [52]. This is to say that patients with hepatic oligo-recurrence from CRC are good candidates for repeat hepatectomy.

The need for the surgical procedure to achieve R0 resection of the tumor while leaving as much future liver remnant as possible is increasing, especially for small CRLMs distant from the hilar plate. Parenchymal-sparing hepatectomy is a widely used, less invasive approach, which generally comprises of wedge resection, minor hepatectomy with parenchymal-sparing approach, and non-anatomical metastasectomy. In addition, segmental resections can be included to parenchymal-sparing hepatectomy in some cases where sufficient liver parenchyma is preserved, or even a right hepatectomy can be considered as parenchymal-sparing hepatectomy if the right liver is full of CRLMs although it is a major hepatectomy [53,54]. A meta-analysis showed that parenchymal-sparing hepatectomy was associated with comparable oncological outcomes, OS, and relapse-free survival (RFS), with better perioperative outcomes and similar incidence of positive surgical margin compared with non-parenchymal-sparing hepatectomy [53]. When limited to a solitary, less than 30 mm, CRLM, parenchymal-sparing hepatectomy was reported to confer greater survival benefit for patients with liver-only recurrence after initial hepatectomy compared to non-parenchymal-sparing hepatectomy because repeat hepatectomy for recurrent CRLMs was more frequently performed in the parenchymal-sparing hepatectomy group than in the non-parenchymal-sparing hepatectomy group (68% versus 24%, *p* < 0.01). Non-parenchymal-sparing hepatectomy was identified as a risk factor of noncandidacy for repeat hepatectomy from multivariate analysis [55]. Therefore, parenchymal-sparing hepatectomy should be considered as much as technically possible rather than non-parenchymal-sparing hepatectomy because of safety, higher rates of resectability of recurrent CRLMs “salvageability”, and better survival, although no formal upper limit regarding number and size of CRLM has been established. A tumor-free margin at least 1 mm wide or 1 cm, if possible, is thought to be necessary for an R0 resection, while some reports showed that neither surgical margin status nor somatic mutations affected the risk of local recurrence after R0-intent hepatectomy for CRLMs. There is no clear consensus regarding the optimal resection margin for good prognostic outcome [56,57,58].

Concurrent resection of hepatic and extrahepatic metastases is associated with worse survival outcomes and higher risk of recurrence than hepatic resection without extrahepatic metastases, and CRLM resection in the presence of concomitant extrahepatic metastases is controversial. However, it is also suggested that concurrent resection may provide the possibility of long-term survival in well-selected patients with oligometastatic CRC [59,60,61].

#### 3.1.2. Percutaneous Ablation

A complete eradication of tumor can be obtained using A0 ablation as well as surgical resection. In the randomized phase II CLOCC trial, which compared systemic therapy plus radiofrequency ablation (RFA) with or without resection versus systemic therapy alone for patients with a median of four CRLMs, an improvement in PFS and in OS for patients receiving RFA was reported [62]; 3-, 5-, and 8-year PFS rates were 27.7%, 24.2%, and 22.3%, respectively, in the combined modality arm versus 11.9%, 5.9%, and 2.0% in the systemic treatment arm, respectively [HR = 0.57 (95% CI: 0.38–0.85); *p* = 0.005]. Likewise, 3-, 5-, and 8-year OS rates were 56.9%, 43.1%, and 35.9%, respectively, in the combined modality arm versus 55.2%, 30.3%, and 8.9% in the systemic treatment arm, respectively [HR = 0.58 (95% CI: 0.38–0.88); *p* = 0.01].

The evidence on the efficacy of ablation is not completely established, but long-term survival rate has been reported to be up to 50% in selected groups such as oligometastatic CRLMs, with various local recurrence rates of 3.6% to 60% [63,64]. Factors like size and location limit the use and effectiveness of percutaneous ablation. Generally, A0 ablation requires a tumor-free margin of >5–10 mm, and the target tumor should not exceed 3 cm [65,66]. As narrower than 5 mm margins and RAS mutation have been reported to be associated with worse local tumor PFS, at least 5-mm margins are recommended for RAS wild-type CRLMs, while over 10-mm margins are necessary for those with RAS mutation to reduce the risk of local recurrence [37,67,68,69]. Although smaller (<3 cm) tumors are more suitable to obtain A0 ablation than larger ones, tumors up to 5 cm may be relatively effectively treated depending on their anatomical position [66,70,71]. Tumors over 5 cm cannot be candidates for percutaneous ablation because of the high rates of local recurrences ranging from 27% to 45% [70,72].

Percutaneous ablation includes several different techniques. Although RFA is the most commonly used modality, with significant data supporting this, microwave ablation can be an alternative of RFA with similar local tumor PFS and morbidity, even a possible better control of perivascular tumors [37]. Irreversible electroporation, which is a nonthermal ablation, could also be recommended for tumors close to blood vessels and bile ducts [73].

Regarding the comparison between surgery and percutaneous ablation as the curative treatment for CRLMs, ablation has been reported to show less morbidity but also shorter survival and higher rates of any type of recurrence [74]. Whether these differences in outcomes are due to limitations and bias of patient selection, lack of operator experience and assessment of safety margin, or ablation technologies remains unclear. A phase III Collision trial is currently ongoing to accurately assess non-inferiority of percutaneous ablation compared to hepatic resection in patients with resectable and ablatable small CRLMs (≤3 cm) [75].

On the other hand, percutaneous ablation is a valid treatment option for patients or lesions for whom resection is unsuitable and for recurrent CRLMs after hepatectomy that can be ablated with clear margins [62,76,77]. Percutaneous ablation in combination with liver resection for eradication of all CRLMs has also been reported to improve perioperative outcomes without compromising long-term survival compared with bilateral resection [78,79]. Because postoperative ablation can avoid the risk of perioperative complication caused by intraoperative ablation without reducing survival, a planned incomplete resection followed by postoperative percutaneous completion ablation of the remaining intentionally untreated lesions may be a safe and effective treatment strategy [80,81].

#### 3.1.3. SBRT

SBRT is a non-invasive external beam radiotherapy that can deliver a high, ablative dose precisely to small, well-defined lesions while minimizing the dose to adjacent normal tissues. A randomized phase II SABR-COMET trial, which compared the impact of additional SBRT on oncological outcomes with standard of care treatment alone in patients with oligo-recurrence in different organs from various primary cancers, including small number of CRC origin (18.2%), demonstrated improvements in OS and PFS [82]. A phase II trial of patients with inoperable CRLMs not amenable to RFA who were treated with SBRT (a total dose of 75 Gy in three consecutive fractions) showed the actuarial 2-year local control rate of 91%. No significantly increased risk of local recurrence was observed in lesions > 3 cm when compared with smaller ones (*p* = 0.92). The actuarial 2-year PFS and 2-year OS were 48% and 65%, respectively [83]. According to a retrospective study for patients with CRLMs treated with SBRT (the prescribed doses of 45–60 Gy in 3–4 fractions), the prescribed dose was a critical factor for local control because the 2-year local control rates significantly differed based on the biologically effective dose of ≤80 Gy, 100–112 Gy, and ≥132 Gy (52%, 83%, and 89%, respectively) [84]. A systematic review showed that SBRT for oligometastatic CRLMs resulted in 2-year local control rate of 59.3% and 2-year OS of 56.5% [85].

Although SBRT is considered an effective and safe treatment, SBRT is an option for patients with CRLMs who are ineligible for surgery because of no randomized controlled trials (RCTs) available comparing each local treatment. However, SBRT has some advantages compared to ablation. Percutaneous ablation is not applicable to the tumors in the invisible area or challenging to reach, such as the subphrenic area or subcapsular location [86]. Ablation is also unsuitable for the tumors adjacent to vasculature due to the potential heat sink effect [87]. On the other hand, SBRT is less affected by these anatomical limitations compared to ablation. Furthermore, SBRT may have a higher local control rate for large tumors (>2–3 cm) compared to ablation [86,88,89]. To be considered suitable for SBRT, lesions should not exceed 5 cm in size, and a 5-mm margin may be necessary to assure a reproducible positioning [90].

### 3.2. Lung Metastases

#### 3.2.1. Surgery

Most of the treatment recommendations for CRLMs can also be applied to lung metastases of CRC. Surgical resection is the preferred method and usually offers 5-year OS of around 50%, reaching up to 70% in selected cases. Even repeat lung metastasectomy can provide a survival benefit, especially for patients with pulmonary oligometastases [91,92,93,94,95,96,97]. Since mCRC limited to the lung are associated with slower growth and prolonged survival, a watchful waiting approach with regular surveillance imaging may also be an alternative strategy [98,99]. Patients with unilateral and a small number of small sized lesions derive better survival, while patients with hilar or mediastinal lymph node metastases, preoperative CEA elevations, shorter disease-free interval, and non-R0 surgery will have poor prognosis [91,92,93,94,95,96,97].

As the goal is to achieve R0 resection while preserving adequate lung function, the first choice for resection of lung metastases is generally wedge or segmental resection of the lung, with a resection margin of 10–20 mm [91,96]. Although pulmonary lobectomy is not recommended due to poor prognosis, it may be performed under rare circumstances such as a deep location of the tumor or massive intraoperative bleeding. For patients with suspected hilar or mediastinal lymph node metastases, lymph node biopsy or dissection can be considered [91].

A multicenter prospective RCT (PUCC-Trial) is now ongoing to investigate the effects of pulmonary metastasectomy in addition to standard medical treatment in comparison to standard medical treatment plus possible local ablative measures such as SBRT for patients with three or more resectable pulmonary metastases from CRC [100].

#### 3.2.2. Percutaneous Ablation

Percutaneous ablation should be considered for patients with lung oligometastases unsuitable for surgery or can be used in combination with surgery for resectable lung metastases. The large prospective database study including 566 patients with 1037 lung oligometastases showed that the survival outcomes after RFA were comparable with those after lung metastasectomy; 5-year OS after RFA ranged from 40.7% to 67.5% depending on risk factors. The challenge of disease control in lung metastatic patients is more linked to the occurrence of new metastases distant from the ablation site than to local recurrences; rate of lung progression was 72.4% at 4 years, while rate of local tumor progression at the site of RFA was 11.0% at 4 years. RFA allows for possible retreatment due to good tolerance and lung parenchyma sparing with no change in respiratory function after RFA. Therefore, 24% of the initially treated patients were retreated by RFA up to four times, resulting in 44.1% 4-year control rate of lung metastatic disease. Because size of tumor was significantly associated with local tumor progression and OS after lung RFA, the tumor should be smaller than 2–3 cm for RFA [36].

Microwave ablation has also been reported to show good outcomes for lung oligometastases of CRC, and no local tumor progression was observed for small tumors ablated with margin of at least 5 mm [101]. Supplementary analysis of a phase II trial (MLCSG-0802) investigated what extent of ablative margin at RFA was required to reduce local tumor progression for CRC lung metastases. The mean ablative margin was 2.7 mm, and an ablative margin of at least 2 mm was important for local control because the rate of local tumor progression was significantly higher when the margin was less than 2 mm (*p* = 0.023) [102]. Therefore, it was suggested that a safety margin of at least 2–5 mm was essential for local control after percutaneous ablation for lung metastases of CRC.

Regarding location of lung metastases, peripheral lesion > 5 cm outer from the pulmonary hilum is appropriate for percutaneous ablation. If the lesion is located in the inner zone or near blood vessels, SBRT should be considered [91,103,104].

#### 3.2.3. SBRT

The efficacy of SBRT for lung metastases from CRC has also been reported, mainly retrospectively. A retrospective study for patients with inoperable lung oligometastases treated with SBRT showed 2-year and 5-year local control rates of 83% and 77%, respectively, regardless of nearly 30% of tumors  ≥  3 cm in size; 2-year and 5-year OS were 69% and 36%, respectively. A biologically effective dose ≥ 100 Gy was independently associated with both better overall survival and local control [105]. The largest retrospective series from 23 centers of 1033 lung oligometastases of CRC treated with SBRT (LaIT-SABR study) reported different 2-year local PFS based on a biologically effective dose of <100 Gy, 100–124 Gy, and ≥125 Gy (76.1%, 70.6%, and 94%, respectively). In the multivariate analysis, a biologically effective dose ≥ 125 Gy significantly reduced the risk of local progression [HR = 0.24 (95% CI: 0.11–0.51); *p* = 0.000]. Furthermore, having lesion > 20 mm and 4–5 simultaneous oligometastases predicted for a polymetastatic evolution [106].

Another large multi-institutional retrospective study investigated 381 oligometastatic CRC lesions treated with SBRT; oligometastases in the lung, liver, and lymph node-soft tissue was 62.7%, 26.5%, and 8.1%, respectively. The 1- and 5-year local recurrence rates were 13.6% and 44.3%, respectively, and a biologically effective dose of ≥120 Gy led to an improvement in local recurrence. The 2- and 5-year OS were 76.1% and 35.9%, respectively. Lung metastases were associated with reduced local recurrence compared to liver metastases [107].

### 3.3. Peritoneal Metastases

Patients with peritoneal metastases are associated with a significantly shorter PFS and OS than those without peritoneal involvement, frequently developing intestinal obstruction [108,109]. The goal of treatment is often palliative rather than curative, and mainly consists of systemic therapy and/or palliative surgery [23]. Therefore, it is controversial whether to include peritoneal metastases of CRC in oligometastases. On the other hand, cytoreductive surgery (CRS) with or without hyperthermic intraperitoneal chemotherapy (HIPEC) has been performed and may be curative in selected patients when carried out at experienced high-volume centers; the 5-year OS can reach 30–50% in patients with limited peritoneal metastases achieving complete resection [110,111,112,113,114,115,116].

The quality of CRS is a very important prognostic factor, and the degree of resection is evaluated as the completeness of the cytoreduction (CC) score. CC-0 indicates no visible residual peritoneal lesions, while CC-1, CC-2, and CC-3 indicate residual tumor < 2.5 mm, 2.5 mm–2.5 cm, and >2.5 cm, respectively [117]. CC-0 provides the best postoperative prognosis, and CC-1 might be effective when combined with HIPEC, while CC-2 and CC-3, considered incomplete, have a negative influence on survival [112,113,114,118,119].

The peritoneal cancer index (PCI) is the most used quantitative method, which estimates the possibility of achieving CC-0 and postoperative prognosis [117]. Increasing PCI is associated with worse survival, and the best survival results are obtained with PCI  ≤ 10, while PCI > 20 is considered as a relative contraindication to CRS due to poor prognosis. The prognosis of PCI 10–20 is different between studies, and these limits are not yet clearly established [112,113,114,116,120,121,122].

According to an RCT comparing CRS and HIPEC with systemic chemotherapy for patients with isolated peritoneal metastases considered resectable preoperatively, increased OS was obtained in patients undergoing CRS with HIPEC [HR = 0.51 (95% CI: 0.27–0.96); *p* = 0.04], although this study inclusion was stopped prematurely due to slow accrual. In addition, regardless of arm and due to the cross-over option, surgical resectability was the main independent determinant for OS [HR = 0.20 (95% CI: 0.09–0.45); *p* = 0.0001] [115].

On the other hand, the efficacy of adding HIPEC to CRS has not been proved in RCTs. A phase III trial (PRODIGE 7) has failed to show the added value of an oxaliplatin-based HIPEC on CRS and perioperative chemotherapy for patients with peritoneal metastases of CRC. This study reported no significant difference in OS, with 5-year OS of 39.4% in the HIPEC group versus 36.7% in the non-HIPEC group [HR = 1.00 (95% CI: 0.63–1.58); *p* = 0.99], but also RFS, with median RFS of 13.1 months in the HIPEC group versus 11.1 months in the non-HIPEC group [HR = 0.91 (95% CI: 0.71–1.15); *p* = 0.43]. In addition, more frequent postoperative late complications were observed in the HIPEC group (26% versus 15%; *p* = 0.035). However, in a post-hoc subgroup analysis, median OS and RFS in patients with a PCI of 11–15 were longer in the HIPEC group than in the non-HIPEC group [HR = 0.44 (95% CI: 0.21–0.9); *p* = 0.021 for OS, and HR = 0.41 (95% CI: 0.21–0.78); *p* = 0.005 for RFS] [116].

Similarly, RCTs (PROPHYLOCHIP-PRODIGE 15 and COLOPEC) have failed to show a survival benefit by addition of prophylactic HIPEC to CRS for patients with high risk of developing peritoneal metastases [123,124].

Therefore, the benefit of HIPEC remains controversial. Although CRS plus HIPEC is considered as an acceptable option, routinely adding HIPEC to CRS is not recommended [23]. There are ongoing trials to investigate if other HIPEC regimens (using mitomycin and different HIPEC procedures) may possibly lead to better outcomes [112,125].

Table 2 shows the summary of local treatments for oligometastatic CRC.

## 4. Multidisciplinary Treatment Strategies with Systemic Therapy

Along with local treatments, systemic therapy is one of the main treatment modalities for mCRC. Although cure of mCRC remains uncommon, more patients can expect prolonged survival with multidisciplinary treatment combining local treatments and systemic therapy, and treatment strategy is crucial to achieve cure, especially for patients with oligometastases.

### 4.1. Systemic Therapy in Metastatic Disease as the Backbone of Multidisciplinary Treatment

Advances in systemic therapy options and efficacy tailored to the molecular and pathologic features of the tumor improved survival of patients with mCRC from 6–12 months to 2–3 years [126].

Standard systemic therapy for advanced or mCRC consists of a two-drug regimen (doublet), such as fluoropyrimidine paired with oxaliplatin (FOLFOX or CAPEOX) or irinotecan (FOLFIRI). Molecular-targeted drugs such as EGFR inhibitors (cetuximab or panitumumab) and bevacizumab are used in combination. Three-drug regimens (triplet), FOLFIRINOX or FOLFOXIRI, are considered for patients with excellent performance status [18,23,25,26].

The frequency of mutations and gene expression patterns of CRC differ depending on the site of the primary tumor, with BRAF V600E mutation, PIK3CA mutation, CpG island methylator phenotype-high, and MSI-H being more frequent in the right colon, and TP53 mutation being more frequent in the left colon [127]. Furthermore, there is increasing evidence that tumors arising from different sides of the colon have distinct prognosis and efficacy of EGFR inhibitors in patients with RAS wild-type CRC.

A combined analysis of six randomized trials (CRYSTAL, FIRE-3, CALGB 80405, PRIME, PEAK, and 20050181) investigated the prognostic and predictive influence of the localization of the primary tumor and the efficacy of addition of EGFR inhibitors compared to bevacizumab or without molecular targeted drugs in patients with RAS wild-type mCRC. The analysis showed a significantly worse prognosis for patients with right-sided tumors compared with those with left-sided tumors for OS, PFS, and objective response rate (ORR) regardless of the choice of molecular targeted drugs. In terms of a predictive effect, a significant benefit for EGFR inhibitors was observed in patients with left-sided tumors [HRs = 0.75 (95% CI: 0.67–0.84) and 0.78 (95% CI: 0.70–0.87) for OS and PFS, respectively] compared with no significant benefit for those with right-sided tumors [HRs = 1.12 (95% CI: 0.87–1.45) and 1.12 (95% CI: 0.87–1.44) for OS and PFS, respectively; *p* value for interaction < 0.001 and 0.002, respectively] [128,129,130,131,132,133,134,135].

In the Japanese prospective phase III trial (PARADIGM) evaluating the superiority of panitumumab vs. bevacizumab in combination with standard doublet first-line chemotherapy (mFOLFOX6) for patients with RAS wild-type mCRC and left-sided primary tumors, panitumumab significantly improved OS compared to bevacizumab [HR = 0.82 (95% CI: 0.68–0.99)]. RR and R0 resection rates were higher with panitumumab [RR = 80.2%; R0 = 18.3%] compared with bevacizumab [RR = 68.6%; R0 = 11.6%] [136,137,138]. Exploratory analysis of patients with right-sided mCRC showed no significant difference in OS between panitumumab and bevacizumab [HR = 1.09 (95% CI: 0.79–1.51)], and the R0 resection rate was similar for patients who received either panitumumab or bevacizumab [137,138]. On the other hand, another phase III trial (CAIRO5) reported that the addition of panitumumab to FOLFOX or FOLFIRI showed no clinical benefit over bevacizumab even in patients with a left-sided and RAS and BRAF V600E wild-type tumor [139]. According to additional evaluation of PARADIGM by circulating tumor DNA (ctDNA), OS tended to be longer for selected patients with no gene alterations treated with panitumumab than for those treated with bevacizumab regardless of tumor sidedness, while OS was similar or inferior with panitumumab vs. bevacizumab irrespective of the primary sidedness in patients with any of gene alterations [140]. Therefore, selection of patients with RAS wild-type mCRC using ctDNA analysis rather than only tumor sidedness may further refine the selection of patients for treatment with panitumumab over bevacizumab in the first-line treatment [25,140].

Currently, major guidelines recommend doublet chemotherapy paired with EGFR inhibitors for RAS/BRAF wild-type left-sided tumors, and doublet or triplet chemotherapy paired with bevacizumab is recommended for right-sided, RAS mutated, or BRAF mutated tumors as the first-line treatment for non MSI-H mCRC [18,23,25,26].

### 4.2. Systemic Therapy in a Multidisciplinary Approach

Adjuvant and neoadjuvant systemic therapy are important components of multidisciplinary treatment with curative intent. To improve oncological outcomes, adjuvant systemic therapy is administered after radical surgery while neoadjuvant systemic therapy is applied before surgery for initially resectable disease. Those given in the perioperative period (before and/or after surgery) are collectively referred to as perioperative systemic therapy. In particular, adjuvant systemic therapy after resection of metastatic disease is sometimes called pseudo-adjuvant therapy. On the other hand, neoadjuvant systemic therapy should be distinguished from preoperative systemic therapy in conversion therapy, which is aimed at patients with initially unresectable disease with the intention of downsizing the tumor burden and ultimately considering resection. Although surgery is the standard local treatment, non-surgical local treatments such as percutaneous ablation and SBRT, as well as surgery, are also combined with systemic therapy to constitute multidisciplinary treatment.

Adjuvant chemotherapy is considered after localized CRC resection, taking into account tumor risk of recurrence, expected benefit from chemotherapy, and risk of complications. For patients with high-risk Stage II or Stage III colorectal cancer, 3–6 months of FOLFOX or CAPEOX or 6 months of capecitabine or 5-FU/leucovorin (LV) are recommended [23,24,26]. The 5-year OS after complete CRLM resection has been reported to be 28–38%, and relapse after resection will occur in almost 75% of the patients, with 5-year RFS ranging from 15% to 35% [141,142,143]. Therefore, perioperative systemic therapy is often administered for patients undergoing liver or lung resection in the setting of resectable mCRC to reduce the risk of recurrence and improve long-term survival. Patients may undergo upfront local treatments, followed by adjuvant therapy. Alternatively, perioperative (neoadjuvant plus adjuvant) systemic therapy can be used [144,145]. Expected advantages of neoadjuvant therapy include earlier treatment of micrometastasis, securement of the safety margin due to tumor shrinkage, determination of responsiveness to the therapy being a strong predictor for prognosis, and avoidance of local treatment for patients with early progression to polymetastatic disease. On the other hand, there is a risk of missing the opportunity for local treatment because of the disease progression or achievement of a complete response during neoadjuvant chemotherapy, thereby making it difficult to identify the lesions [146,147,148]. In fact, it was reported that viable cancer cells were still pathologically present in many of the resected metastatic sites despite achievement of a complete response on imaging. Even among patients with a complete pathological response, long-term remission occurs in only 20–50% of those treated with systemic therapy [148,149,150]. Other possible risks associated with the neoadjuvant chemotherapy include the development of liver steatohepatitis and sinusoidal liver injury caused by irinotecan- and oxaliplatin-based chemotherapies, and morbidity and complications following local treatments [151,152,153,154,155,156]. To reduce the development of these risks, the neoadjuvant period is usually limited to 2–3 months [18,23].

### 4.3. Selected Landmark Evidence from Clinical Trials and Retrospective Studies

#### 4.3.1. Liver Metastases

The optimal perioperative treatment strategy for initially resectable mCRC remains controversial. The phase III EORTC 40,983 study showed that perioperative chemotherapy with FOLFOX4 (6 cycles before and 6 cycles after surgery) increases PFS compared with surgery alone for patients with initially resectable CRLMs, an absolute increase in 3-year PFS of 8.1% (from 28.1% to 36.2%; HR = 0.77 (95% CI: 0.60–1.00); *p* = 0.041). RR after preoperative FOLFOX was 43%, and perioperative mortality was less than 1% in both treatment groups [157]. However, no difference in 5-year OS was observed between the groups (51.2% in the perioperative chemotherapy group versus 47.8% in the surgery-only group; HR = 0.88 (95% CI: 0.68–1.14); *p* = 0.34) [158]. Subgroup analysis suggested that perioperative FOLFOX seemed to benefit, in particular, patients with elevated CEA, that could mean high tumor burden and high tumor activity [159]. Meta-analyses also showed a benefit of perioperative chemotherapy in PFS and disease-free survival (DFS) but not in OS in patients with resectable CRLMs [160,161,162].

With regard to adjuvant chemotherapy, the phase II/III JCOG0603 trial compared hepatectomy alone to hepatectomy followed by 12 courses of adjuvant mFOLFOX6 for patients with liver only mCRC [163]. The 5-year DFS was significantly improved with adjuvant chemotherapy (49.8% versus 38.7%; HR = 0.67 (95% CI: 0.50–0.92); *p* = 0.006). However, 5-year OS was rather higher with hepatectomy alone than hepatectomy followed by chemotherapy (71.2% versus 83.1%; HR = 1.25 (95% CI: 0.78–2.00); *p* = 0.42). Similarly in other RCTs, adjuvant chemotherapy (5-FU/LV or UFT/LV) after CRLM resection prolonged DFS but not OS [164,165]. On the other hand, some meta-analyses have reported OS benefit with the addition of adjuvant chemotherapy in resectable mCRC because each clinical trial might be underpowered to detect smaller differences [166,167]. Although hepatic arterial infusion chemotherapy after CRLM resection has been proved to reduce hepatic recurrences, a survival benefit has not been observed [146,168,169,170].

#### 4.3.2. Metastases Other Than Liver

Few RCTs have been reported examining the benefit of adjuvant therapy after resection of mCRC other than liver metastases. A pooled analysis of two randomized trials (FFCD Trial 9002 and ENG trial) including liver or lung single-site metastases of CRC showed a marginal statistical significance in favor of adjuvant chemotherapy (5-FU/LV) compared to surgery alone [HR = 1.32 (95% CI: 1.00–1.76); *p* = 0.058 for PFS, and HR = 1.32 (95% CI: 0.95–1.82); *p* = 0.095 for OS]. Adjuvant chemotherapy was independently associated with both PFS and OS in multivariable analysis [171]. Imanishi et al. reported a large multicenter retrospective study including 1237 patients at 46 Japanese institutions who underwent surgical resection of lung-limited metastases from CRC. After propensity score matching between patients with and without adjuvant chemotherapy, there were no significant differences between the two groups in terms of DFS [HR = 1.07 (95% CI: 0.82–1.39); *p* = 0.62] and OS [HR = 1.00 (95% CI: 0.69–1.45); *p* = 1.00] [172]. A meta-analysis of 1112 metastasectomies in 927 patients in eight studies also showed no influence of perioperative chemotherapy on survival for patients with pulmonary oligometastases [97]. Despite this, other retrospective studies showed a DFS benefit of adjuvant therapy in patients with pulmonary metastases of CRC [173,174,175].

The CAIRO6 study is a phase II/III randomized trial that investigated perioperative systemic therapy relative to CRS and HIPEC alone for isolated resectable peritoneal CRC metastases. Although perioperative systemic therapy and control arms did not differ regarding the proportions of macroscopic complete CRS-HIPEC (89% versus 86%), objective radiologic and major pathologic RRs to neoadjuvant treatment were 28% and 38% for evaluable patients, respectively. The phase III part, investigating survival outcomes, is still ongoing [176]. An observational cohort study from the Netherlands Cancer Registry investigated the potential benefit of adjuvant systemic chemotherapy for patients with isolated synchronous CRC peritoneal metastases undergoing upfront complete CRS and HIPEC. Adjuvant chemotherapy was associated with improved OS compared with active surveillance (39.2 versus 24.8 months; HR = 0.66 (95% CI: 0.49–0.88); *p* = 0.006) [177]. A systematic review showed that adjuvant chemotherapy, but not preoperative chemotherapy, contributes to improved survival after CRS [178].

Table 3 shows the selected landmark evidence of perioperative systemic therapy for resectable mCRC from clinical trials and retrospective studies.

Thus, it remains unclear whether perioperative chemotherapy improves survival outcomes, especially OS, in patients with resectable mCRC. However, the control or delay of CRC recurrence itself may benefit the patient. Perioperative chemotherapy is not uniformly recommended for initially resectable mCRC and should be decided in consultation with a multidisciplinary team and tumor board, taking into account the potential surgical cure and risk of recurrence. The criterion of multidisciplinary treatment strategy depends on many factors, such as the timing of onset of metastatic disease (synchronous versus metachronous), biology and clinical aggressiveness of the tumor (including RAS, BRAF, or dMMR/MSI mutational status), the history of previous treatments and their respective outcomes, technical criteria for local treatments, the presence of concomitant extrahepatic disease, and the number, size, and location of metastatic sites [18].

### 4.4. Treatment Recommendation

Because many of the primary lesions of CRC can be resected without difficulty regardless of metastatic status, both synchronous and metachronous mCRC is a candidate for curative-intent multidisciplinary treatment if all metastatic lesions are treatable with local treatments, even non-oligometastatic CRC. The intensity of systemic therapy may be different between various factors. Regarding states of oligometastases, oligo-recurrence is considered as a good prognostic state but can also be classified to metachronous oligoprogression or induced oligorecurrence, which may be more aggressive and require more individualized treatment strategies. Treatment for induced oligoprogression and oligopersistence could be considered as conversion therapy for polymetastatic disease.

Previously, local treatments have been indicated only when all metastatic lesions can be eradicated. However, for patients with oligoprogression or oligopersistence, one of the goals of multidisciplinary treatment is eradication of oligometastases with resistance to the current systemic therapy due to tumor heterogeneity by local treatments and then continuation of current systemic therapy, switching to another regimen including maintenance therapy, or interruption of systemic therapy [18,30]. This utility of local treatment is a major clinical achievement resulting from the introduction of the concept of oligoprogression and oligopersistence.

In this section, we introduce multidisciplinary treatments with systemic therapy for oligometastatic CRC based on the perioperative treatment strategies for resectable mCRC. In practice, these treatment strategies may be adaptable to non-surgical local treatments because surgery is combined with other local treatments and systemic therapies in a multidisciplinary approach.

#### 4.4.1. Synchronous Metastases

For patients with resectable synchronous mCRC (mostly liver or lung metastases), the following options are recommended: (1) synchronous or staged colectomy with metastasectomy (bowel-first or liver-first) followed by adjuvant chemotherapy (FOLFOX or CAPEOX > 5-FU/LV or capecitabine); (2) neoadjuvant chemotherapy for 2–3 months (FOLFOX or CAPEOX > FOLFIRI or FOLFIRINOX) followed by synchronous or staged colectomy with metastasectomy, then adjuvant chemotherapy; or (3) colectomy followed by chemotherapy and a staged resection of metastatic lesions, then adjuvant chemotherapy. Generally, the total perioperative treatment duration should not exceed 6 months [18,23].

#### 4.4.2. Metachronous Metastases

Perioperative management of resectable metachronous mCRC differs from that of synchronous disease in terms of the assessment of previous systemic therapy history and the absence of colectomy. For patients without a history of chemotherapy, 6 months of perioperative chemotherapy with oxaliplatin-based regimens is recommended. On the other hand, for patients with a history of previous chemotherapy, especially who relapse during or within 6 months after oxaliplatin-based adjuvant chemotherapy, the optimal individual treatment strategy is required, such as without pre- or postoperative chemotherapy or both of them, considering potential resistance to the regimen and often with a persistent sensitive neuropathy. However, because a disease-free interval of less than 12 months is associated with a poor prognosis, an active regimen for advanced disease may need to be used based on the situation [18,23].

Since the risk of recurrence is clearly higher after resection of metastases than in Stage III, adjuvant therapy with oxaliplatin would be appropriate. However, metastatic surgery may be more invasive than only colectomy of primary tumor and the patient recovers more slowly. Therefore, especially if oxaliplatin has already been used in previous adjuvant chemotherapy or in this neoadjuvant chemotherapy, the omission of oxaliplatin in adjuvant therapy, or in the metachronous setting, observation without adjuvant chemotherapy can be considered [23,26]. Responsiveness to neoadjuvant therapy may also be helpful in planning postoperative therapy [18,23].

Unfavorable oncological criteria, such as synchronous metastases, more than three lesions, bilobar disease, or extrahepatic disease, could lead to the recommendation of neoadjuvant therapy instead of upfront local treatment, especially in an unclear prognostic situation to gain more prognostic insights by observation [18]. Tumor burden and the treatment’s objective (cytoreduction and/or disease control) may affect the choice and intensity of the upfront chemotherapy [18,20,159,181]. However, the magnitude of the benefit provided by chemotherapy intensification remains unclear. Regarding triplet chemotherapy, a pooled analysis of phase III TRIBE and TRIBE2 studies compared upfront FOLFOXIRI plus bevacizumab to doublets (FOLFOX or FOLFIRI) plus bevacizumab for oligometastatic or non-oligometastatic CRC. The triplet significantly improved PFS, OS, and ORR compared to the doublet, independent of the metastatic spread extent or tumor burden [20]. Furthermore, according to an analysis of individual patient data from five trials, triplet plus bevacizumab provided advantages in PFS, ORR, and R0 resection rate at the price of a moderate increase in toxicity [182]. According to NCCN guideline, the triplet regimen is included as an option for neoadjuvant treatment of resectable synchronous mCRC that has high tumor burden [23].

Conversely, patients with favorable oncological criteria (such as metachronous metastases, fewer lesions, unilobar disease, or no extrahepatic disease) that is a similar condition to oligo-recurrence with low tumor burden where cytoreduction and observation of disease aggressiveness are not primary objective of the neoadjuvant chemotherapy would not need an intensified upfront approach. Neoadjuvant or perioperative chemotherapy itself may not be necessary [18,20]. However, it should also be considered that patients with oligometastatic disease and low tumor burden also benefit from intensified neoadjuvant therapy according to a pooled analysis of TRIBE and TRIBE2 [20]. If a preoperative strategy is chosen in such a condition, attention should be paid to the presence of small metastases (10–15 mm), which may disappear while on systemic therapy, with the risk of being missed during surgery [18,23,148]. In such cases, percutaneous fiducial marker placement before preoperative chemotherapy is recommended for accurate localization during resection or ablation of disappeared small CRLMs [183].

### 4.5. Molecular-Targeted Drugs

For patients with initially unresectable RAS wild-type CRLMs, cetuximab in combination with doublet improved the ORR and resectability, and then PFS and OS compared with chemotherapy alone [184]. A meta-analysis of four RCTs also indicated that the addition of EGFR inhibitors to chemotherapy significantly increased the ORR, R0 resection rate and PFS, but not OS [185]. On the other hand, the phase III New EPOC trial aimed to assess the benefit of addition of cetuximab to perioperative chemotherapy for patients with initially resectable RAS wild-type CRLMs, but was closed early because it met protocol-defined futility criteria; PFS was significantly shorter in the cetuximab arm (14.1 versus 20.5 months; HR = 1.48 (95% CI: 1.04–2.12); *p* = 0.030) [186]. A subsequent analysis reported that the addition of cetuximab in the perioperative setting conferred a significant disadvantage in terms of OS (55.4 versus 81.0 months; HR = 1.45 (95% CI: 1.02–2.05); *p* = 0.036) [179].

Similarly, the addition of bevacizumab to doublet or triplet chemotherapy has been reported to improve the ORR, resectability, and survival for patients with initially unresectable mCRC, although some papers do not suggest the benefit [187,188,189,190,191,192]. The phase III HEPATICA study examined adjuvant CAPEOX with or without bevacizumab for patients after radical resection of CRLMs. However, no significant differences seemed to be found in DFS or OS, although conclusions about the effect on survival of additional bevacizumab could not be made because of premature closure of the study [180]. Several RCTs also demonstrated no benefit of the addition of bevacizumab to adjuvant regimens (FOLFOX, CAPEOX, or capecitabine) after resection of stage II and III CRC [193,194,195]. Furthermore, the safety of perioperative administration of bevacizumab has not been adequately evaluated [23,196].

Therefore, perioperative use of EGFR inhibitors or bevacizumab cannot be recommended for patients with initially resectable mCRC [18,23].

### 4.6. Conversion Therapy

Most patients diagnosed with mCRC have little potential of resectability. However, it is important to select patients with initially unresectable disease which can be converted to resectable status after a major response with systemic therapy, that includes induced oligometastases. Survival benefits obtained after resection are reported to be two- to threefold higher than in patients treated with systemic therapy alone, with even a potential of cure [197]. Prolonged systemic therapy-free interval can also be a goal of conversion therapy. The most important indicators for determining resectability of metastases are the likelihood of achieving complete resection with negative surgical margins and maintaining adequate organ reserve [198,199,200,201]. Resection should not be undertaken unless R0 resection of all known metastases is realistically possible, because incomplete resection or only debulking (R1/R2 resection) has not been shown to provide survival benefit [198,202]. Highly active regimen combined with targeted therapy in terms of response rates and early tumor shrinkage is recommended for patients with potentially convertible mCRC because the main objective is not to eradicate micrometastases. Re-evaluation of surgical resectability should be planned 2 months after initiation of systemic therapy, and then every 2 months so that the duration of chemotherapy should be as short as possible, and resection achieved as soon as technically possible in the absence of tumor progression and chemotherapy-induced risks such as liver injury [78,151,155,156,197,203,204,205,206]. Surgery should be performed 3–4 weeks from the previous administration of chemotherapy with or without EGFR inhibitors. Although re-administration of systemic treatment can be considered postoperatively, the total duration of treatment should generally not exceed 6 months. If bevacizumab is administered, at least a 5- to 6-week interval (two half-lives of the drug) between the last dose and surgery should be applied, and re-initiation of bevacizumab should be delayed at least 6–8 weeks postoperatively to reduce the risks such as wound healing complications [18,23].

## 5. Future Directions

The extended studies regarding distinctive molecular profile of oligometastatic CRC have demonstrated that oligometastatic CRC is a specific disease having a potential of cure and not simply an evolutionary step towards polymetastatic disease, which has more aggressive biological behavior [207,208,209,210,211,212]. Loss of KRAS and SMAD4 mutations characterizes the oligometastatic disease while a progressive mutational evolution (gain in KRAS, PI3KCA, BRAF, and SMAD4) is observed in polymetastatic evolving disease. Further exploration of biomarkers characterizing oligometastatic CRC is needed to establish a clinically more promising definition and diagnosis.

In addition, patients with mCRC negative for ctDNA before and/or after surgery were found to have a better prognosis than those positive for ctDNA, and ctDNA status may be useful to predict recurrence early and adjuvant chemotherapy benefits, although some metastatic site such as lung and peritoneum have significantly lower levels of ctDNA, suggesting decreased clinical sensitivity for subclonal variants [213,214,215,216]. Therefore, well-designed prospective trials are essential to optimize treatment strategies in the future, and many clinical trials are now ongoing around world to evaluate adjuvant systemic therapy intensity and the survival outcomes stratified by the status of ctDNA (NCT04680260, NCT05797077, NCT05815082, NCT05062317, NCT05635630, jRCT1071220087).

## 6. Conclusions

mCRC is one of the common causes of cancer related death, and oligometastasis is a positive prognostic factor. The advances in treatments for oligometastatic CRC are crucial for extended survival, and the treatment strategies for individual mCRC patients should be discussed in a multidisciplinary team of experts in the field, considering various oncological factors. Local treatments, such as surgery, percutaneous ablation, and SBRT, and systemic therapy have been the main treatment modalities used to eradicate limited metastatic lesions, but most of the evidence regarding the efficacy and superiority of these modalities, especially local treatments, has been established from retrospective studies due to the lack of RCTs. Since oligometastasis is originally a concept for a clinical status of many solid tumors, with a potential to make a paradigm shift in cancer treatment, it may be difficult to view strictly from the perspective of CRC alone. Therefore, it is important for further developments to understand the comprehensive characteristics and prognosis and to provide a framework for integrated definition, classification, and treatment of the oligometastatic CRC.

## Figures and Tables

**Table 1:** Summary of oligometastatic colorectal cancer classification [16,29].

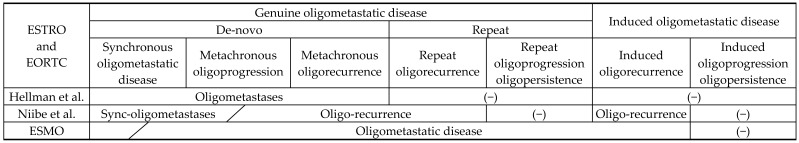

**Table 2:** Summary of local treatments for oligometastatic colorectal cancer.

Local Treatment Modality	Key Points
Surgery	Preferable for resectable lesions R0 resection required while leaving as much organ reserve as possible Repeat liver/lung metastasectomy applicable Surgical approach: -Liver: parenchymal-sparing hepatectomy (wedge resection, non-anatomical metastasectomy, minor hepatectomy) -Lung: edge or segmental resection Safety margin for R0 resection: -Liver: at least 1 mm; 1 cm if possible -Lung: 10–20 mm
Percutaneous ablation	Applicable for non-surgical lesions or in combination with surgery Recurrent CRLMs after hepatectomy are good candidate Preferable lesion size: -Liver: <3 cm preferred; 3–5 cm possible but with higher local recurrence -Lung: <2–3 cm Safety margin for A0 ablation: -Liver: 5 mm for RAS wild type, 10 mm for RAS mutation -Lung: 2–5 mm
SBRT	Applicable for lesions ≤ 5 cm ineligible for surgery 5-mm margin required for a reproducible positioning Biologically effective dose ≥ 100–125 Gy for better local control Suitable lesions than ablation: >2–3 cm in size Location not applicable for ablation (subphrenic, subcapsular, perihilar, adjacent to vasculature)
CRS ± HIPEC	Possible local treatment for peritoneal metastases PCI < 10 preferred; PCI 10–20 controversial CC-0 preferred; CC-1 might be effective Additional HIPEC to CRS should be optional

SBRT: stereotactic body radiotherapy; CRS: cytoreductive surgery; HIPEC: hyperthermic intraperitoneal chemotherapy; CRLM: colorectal liver metastasis; PCI: peritoneal cancer index; CC: completeness of the cytoreduction.

**Table 3:** Selected landmark evidence of perioperative systemic therapy for resectable metastatic colorectal cancer.

Author, Year	N	Study Type	Characteristics	Treatments	Outcomes (95% CI)
Nordlinger 2013 (EORTC 40983) [157,158]	364	RCT Phase III	Resectable CRLMs (≤4) Number: 1–3 (92%) Metachronous onset (65%)	Perioperative FOLFOX4 plus surgery vs. surgery alone	3-year PFS: 36.2% vs. 28.1%; HR = 0.77 (0.60–1.00); *p* = 0.041 5-year OS: 51.2% vs. 47.8%; HR = 0.88 (0.68–1.14); *p* = 0.34
Kanemitsu 2021 (JCOG0603) [163]	300	RCT Phase II/III	R0 resected CRLMs Number: 1–3 (91%) Size (cm): <5 (86%) Metachronous onset (44%)	Surgery plus adjuvant mFOLFOX6 vs. surgery alone	5-year DFS: 49.8% vs. 38.7%; HR = 0.67 (0.50–0.92); *p* = 0.006 5-year OS: 71.2% vs. 83.1%; HR = 1.25 (0.78–2.00); *p* = 0.42
Portier 2006 (FFCD9002) [164]	173	RCT Phase III	R0 resected CRLMs Number: 1–3 (95%) Size (cm): ≤5 (72%) Metachronous onset (71%)	Surgery plus adjuvant 5-FU/LV vs. surgery alone	5-year DFS: 33.5% vs. 26.7%; OR = 0.66 (0.46–0.96); *p* = 0.028 5-year OS: 51.1% vs. 41.1%; OR = 0.73 (0.48–1.10); *p* = 0.13
Hasegawa 2016 [165]	180	RCT Phase III	R0/1 resected CRLMs Number: 1 (46%); mean 3.2 Size (cm): ≤3 (53%); 3–5 (25%) Metachronous onset (55%)	Surgery plus adjuvant UFT/LV vs. surgery alone	3-year RFS: 38.6% vs. 32.3%; HR = 0.56 (0.38–0.83); *p* = 0.003 5-year OS: 66.1% vs. 66.8%; HR = 0.80 (0.48–1.35); *p* = 0.409
Mitry 2008 (FFCD + ENG) [171]	278	Pooled analysis of 2 RCTs	R0 resected liver (94%) or lung (5%) metastases Number: 1 (68%); 2–7 (32%) Metachronous onset (57%)	Surgery plus adjuvant 5-FU/LV vs. surgery alone	5-year DFS: 36.7% vs. 27.7%; HR = 1.32 (1.00–1.76); *p* = 0.058 5-year OS: 52.8% vs. 39.6%; HR = 1.32 (0.95–1.82); *p* = 0.095
Imanishi 2018 [172]	1237	Multicenter retrospective study	R0 resected lung metastases All metachronous onset	Surgery plus adjuvant chemotherapy vs. surgery alone	5-year DFS: 34% vs. 40%; HR = 1.07 (0.82–1.39); *p* = 0.62 5-year OS: 69% vs. 68%; HR = 1.00 (0.69–1.45); *p* = 1.00
Rovers 2020 [177]	393	Retrospective study from cancer registry	Peritoneal metastases Appendiceal tumor excluded Complete CRS + HIPEC Synchronous onset	CRS + HIPEC plus adjuvant systemic therapy vs. CRS + HIPEC alone	5-year OS: 35% vs. 22%; HR = 0.66 (0.49–0.88); *p* = 0.006
Bridgewater 2020 (New EPOC) [179]	257	RCT Phase III	Resectable CRLMs with KRAS wild-type Number: 1–3 (78%) Size (cm): ≤3 (46%) Metachronous onset (37%)	Perioperative chemotherapy + CET plus surgery vs. perioperative chemotherapy alone	PFS: 15.5 vs. 22.2 months; HR = 1.17 (0.87–1.56); *p* = 0.304 OS: 55.4 vs. 81.0 months; HR = 1.45 (1.02–2.05); *p* = 0.036
Snoeren 2017 (HEPATICA) [180]	79	RCT Phase III	R0/1 resected CRLMs Number: 1–3 (81%) Metachronous onset (51%)	Surgery plus adjuvant CAPEOX + BEV vs. Surgery plus adjuvant CAPEOX alone	2-year DFS: 55% vs. 54%; *p* = 0.73 2-year OS: 94% vs. 94%; *p* = 0.43

RCT: randomized controlled trial; CRLM: colorectal liver metastasis; CRS: cytoreductive surgery; HIPEC: hyperthermic intraperitoneal chemotherapy; CET: cetuximab; BEV: bevacizumab; PFS: progression-free survival; OS: overall survival; DFS: disease-free survival; RFS: relapse-free survival.

## Data Availability

The data can be shared up on request.

## Abbreviations

CRC: colorectal cancer; mCRC: metastatic colorectal cancer; CRLM: colorectal liver metastasis; NCCN: National Comprehensive Cancer Network; ESMO: European Society of Medical Oncology; ESTRO: European Society for Radiotherapy and Oncology; EORTC: European Organisation for Research and Treatment of Cancer; ASTRO: American Society for Radiation Oncology; OS: overall survival; SBRT: stereotactic body radiotherapy; PFS: progression-free survival; RFS: relapse-free survival; RFA: radiofrequency ablation; RCT: randomized controlled trial; CRS: cytoreductive surgery; HIPEC: hyperthermic intraperitoneal chemotherapy; CC: completeness of the cytoreduction; PCI: peritoneal cancer index; ORR: objective response rate; ctDNA: circulating tumor DNA; LV: leucovorin; DFS: disease-free survival.
